# Wearing a safety harness during treadmill walking influences lower extremity kinematics mainly through changes in ankle regularity and local stability

**DOI:** 10.1186/1743-0003-9-8

**Published:** 2012-02-03

**Authors:** Leslie M Decker, Fabien Cignetti, Nicholas Stergiou

**Affiliations:** 1 Nebraska Biomechanics Core Facility, University of Nebraska at Omaha, 6001 Dodge Street, Omaha, NE 68182-0216, USA; 2 Institut des Sciences du Mouvement Etienne-Jules Marey, UMR 7287 CNRS & Aix-Marseille Université, 163 avenue de Luminy, Case Postale 910, 13288 Marseille Cedex 9, France; 3 Environmental, Agricultural and Occupational Health Sciences, College of Public Health, University of Nebraska Medical Center, 985450 Nebraska Medical Center, Omaha, NE 68198-5450, USA

## Abstract

**Background:**

Wearing a harness during treadmill walking ensures the subject's safety and is common practice in biomedical engineering research. However, the extent to which such practice influences gait is unknown. This study investigated harness-related changes in gait patterns, as evaluated from lower extremity kinematics during treadmill walking.

**Findings:**

Healthy subjects (*n *= 10) walked on a treadmill at their preferred speed for 3 minutes with and without wearing a harness (LiteGait^®^, Mobility Research, Inc.). In the former condition, no weight support was provided to the subjects. Lower extremity kinematics was assessed in the sagittal plane from the mean (mean_RoM_), standard deviation (SD_RoM_) and coefficient of variation (CoV_RoM_) of the hip, knee, and ankle ranges of motion (RoM), as well as from the sample entropy (SampEn) and the largest Lyapunov exponent (LyE) of the joints' angles. Wearing the harness increased the mean_RoM _of the hip, the SD_RoM _and the CoV_RoM _of the knee, and the SampEn and the LyE of the ankle. In particular, the harness effect sizes for both the SampEn and the LyE of the ankle were large, likely reflecting a meaningful decline in the neuromuscular stabilizing control of this joint.

**Conclusions:**

Wearing a harness during treadmill walking marginally influences lower extremity kinematics, resulting in more or less subtle changes in certain kinematic variables. However, in cases where differences in gait patterns would be expressed through modifications in these variables, having subjects walk with a harness may mask or reinforce such differences.

## Findings

Treadmill walking is commonly used for biomedical engineering research and rehabilitation purposes. In research, it allows investigators to acquire gait variables from a large number of consecutive steps in an easy and time-saving manner [1]. In rehabilitation, when combined with a body weight support (BWS) system, it enables patients who are unable to fully bear their weight to safely initiate a retraining program [2]. Investigations have thus examined whether gait patterns are equivalent between treadmill and overground walking [3,4], and kept normal while walking with a BWS system [5,6]. Overall, treadmill and overground walking have been shown to be mechanically similar [3], but with reduced variability and improved local stability of the lower extremities during treadmill walking [4,5]. On the other hand, increasing the level of BWS has been found to progressively alter gait kinematics and kinetics [6,7], with more pronounced changes in the kinetic patterns [7].

However, an issue related to treadmill walking that has been poorly investigated concerns the effect of the harness alone (0% BWS) on gait. Ivanenko et al. [7] indicated that the kinematic and muscle activity patterns of the lower extremities during treadmill walking with a harness at 0% BWS were roughly similar to previous findings they obtained using a setup where subjects walked on a treadmill without harness [8]. Although these findings provide indirect evidence that the impact of the harness alone on gait patterns is likely limited, no definite conclusion can be drawn since the effect of wearing a harness was not examined *per se*. Recently, Aaslund and Moe-Nilssen [9] specifically investigated the effect of wearing a harness on trunk movements during gait and demonstrated a reduction in the vertical trunk acceleration. Since damping of vertical trunk acceleration is predominantly achieved by the lower extremities, the authors suggested that significant modulations of lower extremity control may occur when wearing a harness. Therefore, our study aimed to explore this issue. We hypothesized that wearing a harness would be associated with significant changes in gait patterns, as evaluated from lower extremity kinematics.

**Figure 1:**
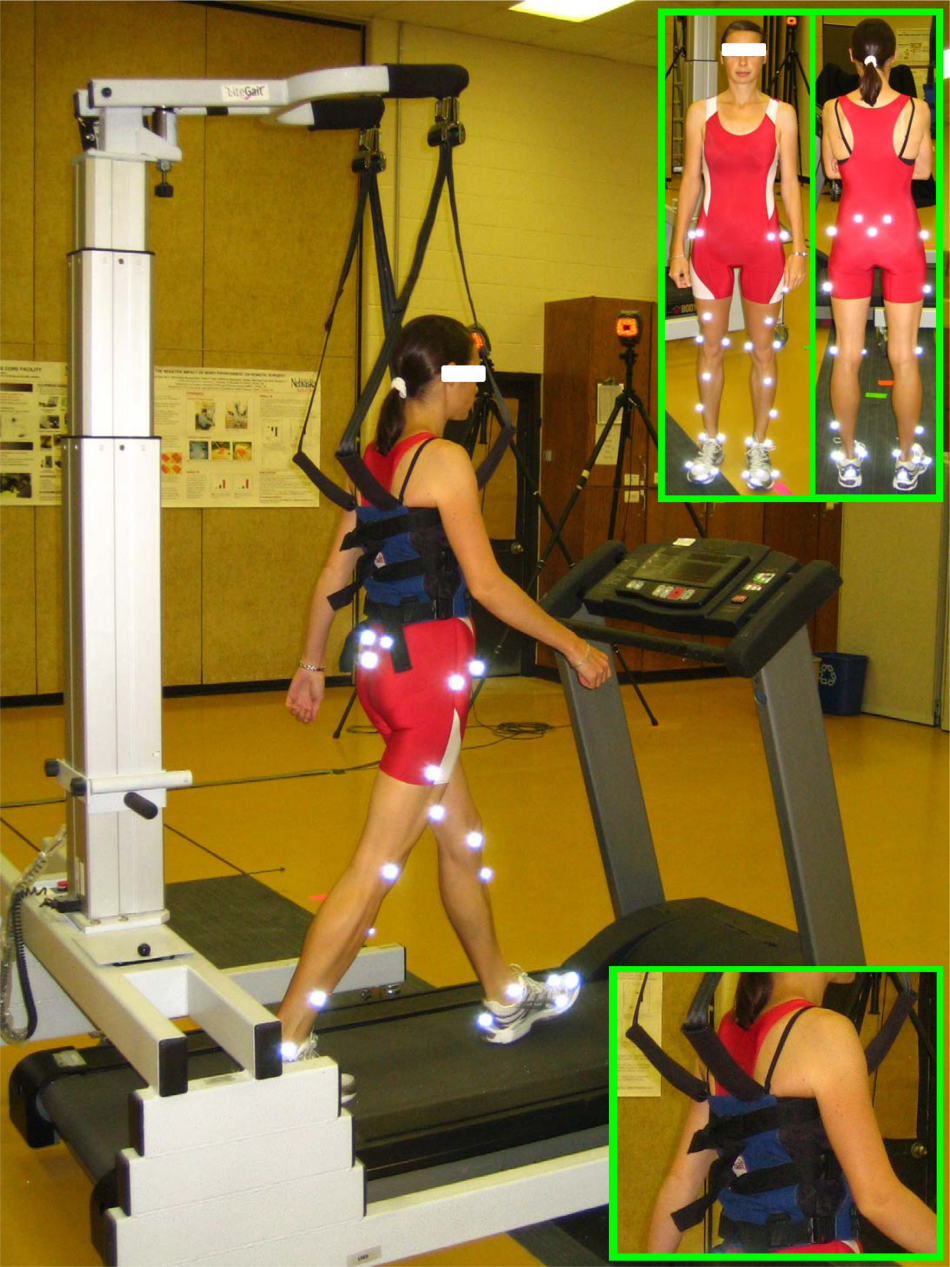
**Experimental set-up with a subject fitted into the LiteGait^® ^system (Mobility Research, Inc., Tempe, AZ)**. This safety system consists of a lightweight waist harness straps linked to a telescoping metallic arm. The metallic arm was adjusted based on the subject's height so that the system did not provide any body weight support (slack straps). The absence of support was also monitored using the BiSym digital microprocessor of the LiteGait^® ^system. The harness size (small, medium and large) was selected based on the subject's upper body dimensions. It was tightened using locking straps located in the subject's back based on two criteria: (i) the subject had to feel comfortable wearing the harness while walking, and (ii) the harness had to fit well the waist without moving around it during walking. Reflective markers were attached to anatomical landmarks on the lower extremities, including the anterior and posterior superior iliac spine, lumbosacral joint, greater trochanter of the femur, lateral mid-thigh, front lower thigh, lateral and medial epicondyles of the femur, front mid-shank, lateral lower shank, lateral and medial malleoli, lateral border of the fifth metatarsal head, medial border of the first metatarsal head, lateral and medial processes of the calcaneal tuberosity, heel, and between the second and third metatarsophalangeal joints.

Ten healthy right leg dominant subjects (4 females and 6 males; age: 24.8 ± 4.0 years; body weight: 80.5 ± 18.41 kg; height: 1.78 ± 0.10 m) participated in the study after signing an institutionally approved informed consent form. The subjects were free of lower extremity injuries and disabilities that might influence walking ability. Reflective markers were attached to a tight fitting suit at specific anatomical landmarks on the lower extremities [10,11] (Figure 1). After having determined the subjects' preferred walking speed (PWS; mean ± SD of the group: 1.11 ± 0.16 m.s^-1^) on treadmill (312-C, Bodyguard) using a well-established protocol [12], two treadmill walking conditions, presented randomly, were performed at PWS: wearing and without wearing a harness. For the harness condition, participants were fitted into the LiteGait^® ^partial weight bearing system (Mobility Research, Inc.) (Figure 1). The system was adjusted to not support the subject (0% BWS), as measured and monitored using the BiSym, a digital microprocessor that displays in real-time the load support provided by the LiteGait^®^. For each condition, subjects walked for 3 minutes.

The three-dimensional marker positions were acquired (60 Hz) with an 8-camera Motion Analysis Eagle Digital system. The anatomical joint angles of the right/left hips, knees and ankles were then obtained in the sagittal plane using published algorithms [11]. Only this plane of motion was considered since data from the other planes collected via skin markers are associated with increased measurement error [13]. The ranges of motion (RoM) were then identified, from the 3 minute time series, by subtracting the minimum joint angle from the maximum joint angle for each gait cycle. A gait cycle corresponded to the interval between consecutive ipsilateral toe-off events, with the toe-off defined from the maximum backward displacements of the marker located between the second and third metatarsophalangeal joints. For consistency across subjects, the RoM time series were shortened to 134 data points, which was the number of gait cycles of the slowest subject.

**Figure 2:**
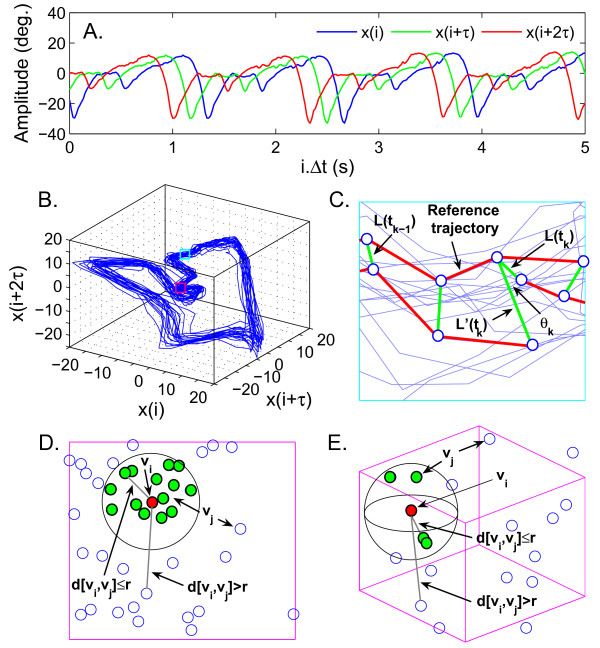
**Attractor reconstruction and calculation of the largest Lyapunov exponent (LyE) and sample entropy (SampEn)**. (A) The original $x{\left(i\right)}_{i=1}^{N}$ angle time series and the time-delayed copies [*x*(*i*+ *τ*),...,*x*(*i *+ (*m*-1)*τ*)] used for attractor reconstruction. (B) The attractors were composed of sets of *m*-dimensional vectors *v*(*i*) = [*x*(*i*), *x*(*i*+ *τ*),...,*x*(*i *+ (*m*-1)*τ*)], with *i *= 1,...,*N *- (*m*-1)*τ*. The delay *τ *was obtained from the first minimum of the average mutual information function and the dimension *m *was selected where the percentage of the global false nearest neighbours approached zero. (C) The *LyE *algorithm tracked the divergence of nearest neighbours over time, focusing on a reference trajectory with a single nearest neighbour being followed and replaced when its separation L'(*t_k_*) from the reference trajectory becomes large. The new neighbour was chosen to minimize the replacement length L(*t_k_*) and the angular separation *θ_k_*. Once the reference trajectory has gone over the data sample, $LyE={\left(t_{M}-t_{0}\right)}^{-1}\sum_{k=1}^{M}\text{log}\left[L^{\prime }\left(t_{k}\right)/L\left(t_{k-1}\right)\right]$ was estimated, with *M *the total number of replacement steps [14,15]. (D) For the *SampEn*, the first step consisted in calculating $C_{i}^{m}\left(\tau ,r\right)={\left(N-m\tau \right)}^{-1}\left(\text{number of }j\text{ such that }d\left[v\left(i\right),v\left(j\right)\right]\leq r\right)$, where *j*≠*i *ranges from 1 to *N *- *mτ*, and $d\left[v\left(i\right),v\left(j\right)\right]=\underset{0\leq k\leq m-1}{\text{max}}\left(|x\left(j+k\right)-x\left(i+k\right)|\right)$ is the maximum difference between the scalar components of the vectors [*v*(*i*), *v*(*j*)]. The distance *r *was chosen as 0.2× standard deviation of *x*(*i*). The density $\Phi^{m}\left(\tau ,r\right)={\left(N-m\tau \right)}^{-1}\sum_{i=1}^{N-m\tau }C_{i}^{m}\left(\tau ,r\right)$ was obtained afterwards. (E) The procedure was repeated for an (*m*+1)-dimensional attractor, by computing Φ^*m*+1^(*τ, r*). Finally, the negative log likelihood of the conditional probability that two close vectors (within *r*) in a *m*-dimensional attractor remain close in a (*m*+1)-dimensional attractor was obtained as *SampEn *= -τ^-1 ^log (Φ^*m*+1^(*τ, r*)/Φ*^m^*(*τ, r*)) [16].

Gait function was examined from the joint RoM time series by calculating the mean (mean_RoM_), the standard deviation (SD_RoM_) and the coefficient of variation (CoV_RoM_). From the time series of joint angles were also obtained the largest Lyapunov exponent (LyE) and the sample entropy (SampEn), quantifying local stability and regularity of the joint kinematic patterns, respectively. Smaller LyE and SampEn values reflect more stable and periodic patterns, while larger values reflect more unstable and irregular behaviours, respectively. Details on the algorithms and input parameters used to calculate these measures are provided in Figure 2 and available elsewhere [14-16]. For each joint, the above measures were analyzed using two-way within-subjects ANOVAs (Side: Right/Left; Harness: With/Without). The factor side was included into the analyses to evaluate possible changes in the (a)symmetrical behaviour of the lower extremities due to the harness (a possible side×harness interaction effect). Effect sizes are reported as *η*^2 ^= SS_explained_/SS_total_.

**Figure 3:**
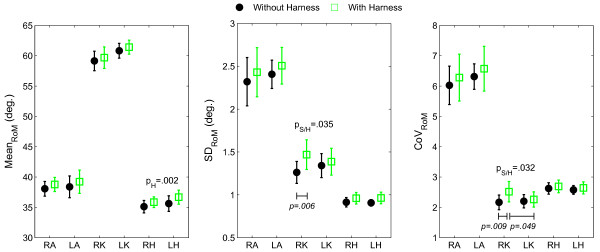
**Linear measures from the lower extremity joint range of motions**. Mean_RoM_: central tendency, SD_RoM_: standard deviation, and CoV_RoM_: coefficient of variation, for the range of motion (RoM) of all three joints of the lower extremities. LA: left ankle. RA: right ankle. LK: left knee. RK: right knee. LH: left hip. RH: right hip. Error bars denote between-subjects standard error of the mean. Statistically significant effects in the two-way (Side: Right/Left; Harness: With/Without) repeated measures ANOVAs are reported, with *p*_H _and *p*_S/H _corresponding to *p*-values for the harness main effect and the side×harness interaction effect, respectively. Results from the post-hoc Tukey's HSD analyses are reported in the presence of an interaction effect, with the difference between average values indicated with horizontal bars and *p*-values.

Only three out of fifteen ANOVAs revealed a significant harness main effect and two out of fifteen a side×harness interaction effect. For mean_RoM_, a significant harness effect (*F*[1,9] = 18.17; *p *= 0.002; *η*^2 ^= 0.18) was observed at the hip, with the harness producing a larger value (36.27 ± 1.15° vs. 35.26 ± 1.02°, Figure 3). For SD_RoM _and CoV_RoM_, significant interaction effects (*F*[1,9] = 6.12; *p *= 0.035; *η*^2 ^= 0.06; and *F*[1,9] = 6.37; *p *= 0.032, *η*^2 ^= 0.06, respectively) were observed at the knee, with the harness causing larger values at the right knee only (SD_RoM_: 1.46 ± 0.17° vs. 1.26 ± 0.12°, CoV_RoM_: 2.51 ± 0.33% vs. 2.16 ± 0.24%, Figure 3). Besides, with harness, the right knee exhibited larger CoV_RoM _values than its left counterpart (2.51 ± 0.33% vs. 2.26 ± 0.25%, Figure 3).

**Figure 4:**
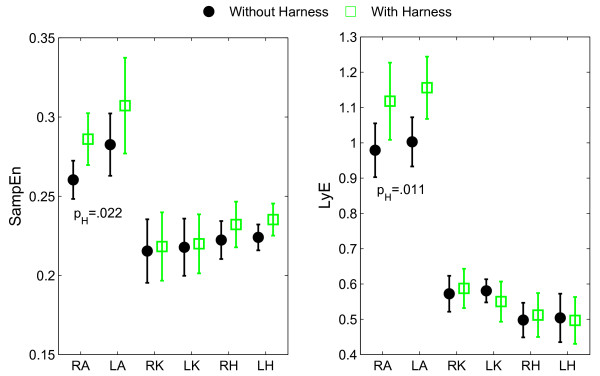
**Nonlinear measures from the lower extremity joint angles**. SampEn: Sample Entropy. LyE: largest Lyapunov exponent. LA: left ankle. RA: right ankle. LK: left knee. RK: right knee. LH: left hip. RH: right hip. Statistically significant effects in the two-way (Side: Right/Left; Harness: With/Without) repeated measures ANOVAs are reported, with *p*_H _the *p*-value for the harness main effect.

Finally, a significant harness effect was found for both the SampEn and the LyE of the ankle (*F*[1,9] = 7.59; *p *= 0.022; *η*^2 ^= 0.14; and *F*[1,9] = 9.99; *p *= 0.011, *η*^2 ^= 0.31, respectively), with larger values with harness (SampEn: 0.29 ± 0.02 vs. 0.27 ± 0.01; LyE: 1.13 ± 0.09 vs. 0.99 ± 0.07; Figure 4).

Overall, the impact of the harness on lower extremity kinematics has been found to be limited, with most of the measures being similar between the two conditions (with and without harness). However, more or less subtle differences in kinematics have also been observed and deserve to be discussed. First, the increased hip RoM (~1°) with harness indicates a small deviation from normal gait. It also confirms that the LiteGait^® ^system was properly adjusted and did not support the subjects since any BWS leads to decreased joint RoM [6]. Increased hip RoM in the sagittal plane has been observed when carrying external loads [17,18], which compensates for the decreased pelvic rotation so that walking speed is kept constant [18]. Although the extra load from the harness is negligible in our experiment, the contact of the harness to the waist may have reduced transverse pelvic rotation that was compensated by increasing hip RoM. This assumption calls for examining the effects of the harness on pelvic rotation that together with thoracic rotation and arm movements critically determine trunk momentum and gait stability [19,20]. However, it is important to not over-enhance this result since a one-degree increase in hip RoM is limited, especially when balanced with measurement errors of kinematic data and intra-subject variability in gait performance. Second, the larger variability observed for the right knee motion with the harness, through increased SD_RoM _(~0.2°) and CoV_RoM _(~0.4%), also supports the hypothesis and can be interpreted as a decline in the functional ability to walk [4,21,22]. The fact that differences were only observed at the right knee may originate from gait asymmetry [23,24], and specifically, from functional differences between the lower extremities; the dominant and non-dominant (here, right and left, respectively) lower extremities being mainly responsible for propulsion and support/control, respectively [23]. In this scenario, the right lower extremity is possibly less tightly controlled, so that the harness effect is magnified on it. However, the size of the harness effect on knee variability (*η*^2 ^= 0.06) was small, questioning the real meaning of this effect in terms of walking functionality. Third, the higher LyE and SampEn values noticed at the ankle indicated a declined (more random-like) control of this joint when walking with the harness. The increased LyE also indicated a greater local instability of this joint [16]. Therefore, there is plausibly a negative impact of the harness on the neuromuscular control of the ankle, especially in view of the large harness effect sizes on the LyE and the SampEn (*η*^2 ^= 0.31 and 0.14, respectively). Change in control was restricted to the ankle possibly because the greater inertias of the proximal joints attenuated the effect of the perturbation (here, arising from the harness) on their motions, so that their regularity and local stability remained unchanged [25].

In summary, it is reasonable to conclude that securing a subject in a harness during walking subtly affects lower extremity kinematics, with more important changes at the ankle. In cases where differences in gait control would be expressed through modifications in lower extremity kinematics, such differences may be masked or reinforced by having subjects walk with a harness.

## Competing interests

The authors declare that they have no competing interests.

## Authors' contributions

LD designed the study and carried out the experiment. Both LD and FC analyzed the data, interpreted the results, drafted and revised the manuscript. FC conceived all figures. NS assisted in drafting and revising the manuscript. All authors approved the final version of the manuscript.
